# Added value of adjuvant chemotherapy in patients with node-positive pT1-2 colon cancer: a national SNAPSHOT analysis

**DOI:** 10.1093/oncolo/oyag171

**Published:** 2026-05-13

**Authors:** Julia Hanevelt, Jan W B de Groot, Eva Rademaker, Bobby Zamaray, Richard M Brohet, Leon M G Moons, Frank P Vleggaar, Esther C J Consten, Pieter J Tanis, Wouter H de Vos tot Nederveen Cappel, Henderik L van Westreenen, the Dutch Snapshot Research Group (DSRG), Dutch Complex Colon Cancer Initiative (DCCCI) and Collaborators Snapshot Registry, D C Van der Aa, S M Van Aalten, I Aanen, F J Amelung, P Van Amstel, E Ancion, J J Atema, T S Aukema, V Baart, E Z Barsom, V P Bastiaenen, T Bazuin, M A J Becker, H J Belgers, V M Belvroy, R H A Berndsen, B Biersteker, J D W Van der Bilt, J G Bloemen, S Bluiminck, F C Den Boer, , E G Boerma, M C Boonstra, W A A Borstlap, L A Brugts, J W A Burger, T A Burghgraef, S M M De Castro, M Cleveringa, R J S Coelen, J W T Dekker, A Demirkiran, E Dijkema, S Dijkink, S A Dingemans, L Van Dullemen, E B Van Duyn, A J Eleveld, H A Galema, E G M Van Geffen, R T J Geitenbeek, A A W Van Geloven, A H C Gielen, C A Gispen, M J P M Govaert, W M U Van Grevenstein, B A Grotenhuis, A A J Grüter, S F Hardon, A G Den Hartog, K Havenga, J A G Van der Heijden, F Heilijgers, T B M Van den Heuvel, R S Hijmans, J Van Hilst, I Hochstenbach, C Hoff, J C Hol, S Janki, A C H M Jongen, I Kappers, S Karhof, B A J Kertzman, M M Kieboom, N Koemans, J L M Konsten, W H Kopp, O W Kranenburg, P Krielen, A Kumas, M Kusters, D P V Lambrichts, B Lamme, G L Van Leeuwen, J W A Leijtens, E W Lockhorst, H Lutfi, S Malm, H T J Mantel, G A Martini, P Meijer, J Melenhorst, D M Mens, M J H Metman, L R Moolenaar, L C F De Nes, J A Nieuwstraten, J Nonner, S E Van Oostendorp, S J Oosterling, K C M J Peeters, C M W Pesch, C M L Peters, M W H Philipsen, W Y Van der Plas, M M Poelman, F Polat, A E Posma-Bouman, B B Pultrum, J M Van Rees, W P Reinders, P R De Reuver, M C Richir, T W H Rijnhout, M M Romeijn, D Roorda, C C Van Rossem, J Rothbarth, M Ruig, H G Sahin, I Said, R A Schasfoort, J Scholten, J M J Schreinemakers, P M E Schuivens, D Schweitzer, C Sietses, M Sietzema, G D Slooter, N Smakman, B P Smalbroek, A B Smits, E J Spillenaar Bilgen, E J A Steller, T F Stoop, M Straver, S K Stuart, A Suntharan, A K Talsma, K Trumpi, J B Tuynman, J Veenhuizen, A W H Van de Ven, E G G Verdaasdonk, P M Verheijen, A Villerius-Meijer, M Vissers, E L K Voogt, R P H De Vries, S T Van Vugt, D D De Waard, T R Wagner, A K Warps, R H A Welling, M Westerterp, J K Wiggers, A R Wijsmuller, C D M Witjes, N Van Zenden, D D E Zimmerman, S J M Zomer, E S Zwanenburg, E S Zwart, S L M Zwetsloot, I H J T de Hingh, N F M Kok, M J Lahaye, O W Kranenburg, A G J Aalbers, P Snaebjornsson

**Affiliations:** Department of Gastroenterology and Hepatology, Isala, Zwolle, 8025, The Netherlands; Department of Medical Oncology, Isala, Zwolle, 8025, The Netherlands; Department of Surgery, Isala, Zwolle, 8025, The Netherlands; Department of Surgical Oncology and Gastrointestinal Surgery, Erasmus MC Cancer Institute, Rotterdam, 3062, The Netherlands; Department of Surgery, Isala, Zwolle, 8025, The Netherlands; Department of Epidemiology & Statistics, Isala, 8025, The Netherlands; Department of Gastroenterology and Hepatology, University Medical Center Utrecht (UMCU), Utrecht, 3584, The Netherlands; Department of Gastroenterology and Hepatology, University Medical Center Utrecht (UMCU), Utrecht, 3584, The Netherlands; Department of Surgery, Meander Medisch Centrum, Amersfoort, 3813, The Netherlands; Department of Surgery, University Medical Center Groningen, Groningen, 9713, The Netherlands; Department of Surgical Oncology and Gastrointestinal Surgery, Erasmus MC Cancer Institute, Rotterdam, 3062, The Netherlands; Department of Gastroenterology and Hepatology, Isala, Zwolle, 8025, The Netherlands; Department of Surgery, Isala, Zwolle, 8025, The Netherlands

**Keywords:** adjuvant chemotherapy, early-stage colon cancer, T1 colorectal cancer, T2 colorectal cancer, lymph node metastases, colon cancer

## Abstract

**Background:**

Patients with node-positive colon carcinoma commonly receive adjuvant chemotherapy, regardless the tumor’s T-stage. However, early-stage tumors (pT1-2 CC) are largely underrepresented in landmark studies supporting this treatment. This study evaluates the application of adjuvant chemotherapy in those patients based on daily practice.

**Patients and methods:**

Patients who underwent surgery for either pT1- or pT2-CC were identified from the nationwide SNAPSHOT database and stratified by age (<75 or ≥75 years). Competing risk regression and (cause-specific) Cox proportional hazard models identified factors associated with 5-year cumulative incidence of recurrence and overall survival (OS), respectively.

**Results:**

Lymph node metastases were found in 381 out of 2,312 (16.5%) patients, of whom 275 (72.2%) received adjuvant chemotherapy. The cumulative incidence of recurrence was 0.09 (95% CI 0.06-0.12) and 0.18 (95% CI 0.11-0.27) in patients who did or did not receive adjuvant chemotherapy, respectively (*P* = .007). In patients under 75, adjuvant chemotherapy was associated with significantly higher OS (91.3% vs 68.1%, *P* < .001). Corresponding OS probabilities in elderly patients (≥75 years) were 84.5% vs 55.1%, *P* = .003. After adjusting for confounding, this difference remained only significant in patients under 75: HR_adj_ 0.5, 95% CI 0.1-0.7 and HR_adj_ 0.5, 95% CI 0.2-1.3, respectively. The recurrence rate was not significantly different between patients receiving capecitabine/oxaliplatin (CapOx) and those on Capecitabine monotherapy (CIF 0.09, 95% CI 0.06-0.14 vs 0.05, 95% CI 0.01-0.16, *P* = .49).

**Conclusion:**

Adjuvant chemotherapy is associated with reduced risk of recurrence in patients with node-positive pT1-2 CC. Advantages on OS could not be demonstrated in elderly pT1-2N1-2 patients.

Implications for Practice This study evaluated the effectiveness of adjuvant chemotherapy in patients with node-positive pT1-2 colon cancer using real-world data. While adjuvant chemotherapy was associated with a reduced risk of disease recurrence, its benefit on overall survival was limited to those under 75 years of age. Given the weak evidence base, particularly for elderly patients, treatment decisions should be individualized, taking into account patient age, comorbidities, and overall prognosis. As conducting large randomized controlled trials in this patient subgroup is infeasible, clinicians should support the collection and use of robust real-world data, including national audits and registries, to inform future guidelines.

## Introduction

According to the current standard of care and national guideline, all patients diagnosed with stage III colon carcinoma (CC) are advised to undergo adjuvant chemotherapy with either fluoropyrimidine monotherapy or the combination of oxaliplatin and a fluoropyrimidine (FOLFOX or CapOx), regardless of the depth of tumor invasion (T-stage).^1–3^ However, the large randomized clinical trials that provided the evidence for the efficacy of adjuvant chemotherapy and its regimens were primarily based on patients with advanced T-stage. The vast majority of the study populations of these trials consisted of pT3 tumors. Patients with pT1 or pT2 tumors were either minimally represented (4%-15%) or not represented at all, which raises questions about the strength of evidence for adjuvant chemotherapy in patients with low T-categories.^4–6^ Additionally, T1-2 carcinomas may differ from more advanced tumors in both their biological behavior and genetic profile, which may modulate chemosensitivity. For example, data from a recent pooled analysis of the ACCENT trial indicated that oxaliplatin-based adjuvant therapy improved survival in pT3 patients, while no significant benefit was seen in those with pT1-2 tumors.^7^

Several small cohort studies addressed this lacuna with conflicting results. A recent single-center retrospective study by Pian et al. suggested that adjuvant chemotherapy is not beneficial in patients with pT1-T2 N1 CC patients (stage IIIa disease) who underwent curative surgery.^8^ Although the number of patients included was small (*n* = 41), both overall survival and disease-free survival were comparable between the patients who received chemotherapy (FOLFOX) and those who did not.^8^ Their findings are consistent with studies of Kim et al. and Park et al., who showed that adjuvant chemotherapy did not affect disease-free survival in patients with IIIa disease as well; however, overall survival outcomes were not reported.^9^^,^^10^ Yan et al. and Ganapathi et al. studied much larger cohorts of pT1 CC patients with positive lymph nodes specifically. In contrast to the earlier mentioned studies, adjuvant chemotherapy reduced CRC-related mortality by 33.9% and improved overall survival by 20.4% after propensity matching.^11^^,^^12^

Besides limited representation of early T-categories in landmark studies on adjuvant chemotherapy, these studies also excluded elderly patients (ie, patients over 75 years of age).^4^ However, with almost half of CC patients being over the age of 75 years at diagnosis, there is lack of evidence in a substantial subgroup as extrapolation from study findings to those patients is questionable.^13^

As adjuvant chemotherapy, particularly oxaliplatin, is often associated with severe toxicity, it is essential to be informed about its impact on survival in both younger and older patients.^14^ Therefore, the primary aim of this study was to evaluate the added value of adjuvant chemotherapy in younger and older patients with lymph node-positive pT1-T2 colon cancer.

## Materials and methods

A retrospective nationwide cross-sectional cohort study was conducted, including patients who underwent curatively intended surgery for pT1 or pT2 CC between January 1, 2014, and December 31, 2015. These patients were retrieved from the national collaborative SNAPSHOT colon cancer database of the Dutch Snapshot Research Group. Patients in this cohort were originally identified through the Dutch ColoRectal Audit (DCRA), a prospective mandatory national clinical registry of all patients undergoing surgery for primary CRC.^15^ Due to the regulatory mandate by the Inspectorate of Healthcare to evaluate the quality of care, patients are not required to provide informed consent for data collection. The DCRA data were supplemented with additional detailed information, including long-term oncological follow-up data, from original patient files by local collaborators in 50 hospitals in the Netherlands. Data collection was performed by surgical residents using a standardized case report form and was closely supervised and validated by a consultant gastrointestinal, ensuring consistency and accuracy across participating centers.

Exclusion criteria were (1) synchronous second primary or metachronous CC (within 5 years of primary diagnosis), (2) synchronous metastases, (3) any form of neo-adjuvant therapy, (4) death ≤30 days after surgery, (5) appendiceal cancer, (6) tumor-related complications (proximal perforation, tumor perforation, or peritumoral abscess), or (7) clinical suspicion of ingrowth into surrounding structures or organs (cT4) despite final pathological examination showed pT1-2.

### Outcomes and definitions

Tumor location was classified as either right colon (including the cecum, ascending colon, hepatic flexure, transverse colon, and splenic flexure) or left colon (comprising the descending colon and sigmoid colon). Postoperative complications were defined as any deviation from the normal recovery process within 30 days following surgery. A re-intervention referred to any surgical, endoscopic, or radiological procedure performed within 30 days from index surgery. Tumor differentiation was categorized into good/moderately differentiated and poorly differentiated, based on the WHO classification of tumors of the digestive system.^16^ The lymph node ratio, calculated as the number of positive lymph nodes divided by the total number of removed lymph nodes, was transformed from a continuous variable into a binary variable based on the cohort’s median lymph node ratio (< 0.1 or ≥ 0.1). Overall survival (OS) was defined as the time from surgery to death from any cause or the date of last follow-up. Cumulative incidence of recurrence (CIF) was measured from the time of surgery until the first occurrence of recurrent disease (locoregional recurrence or distant metastasis), considering death from any cause as a competing event. Loco regional recurrence was defined as any intra-abdominal recurrence, including peritoneal recurrences, but excluding distant metastases in the liver. Patients were considered lost to follow-up if follow-up data were missing or if the follow-up period was less than 3 months.

### Statistical analysis

Mean and standard deviation were used for normally distributed continuous data, whereas median and interquartile range (IQR) were used for non-normally distributed continuous data. Categorical data were summarized with frequencies and proportions. The baseline characteristics of the chemotherapy groups were compared to those who did not receive chemotherapy, and stratified by age (<75 or ≥75 years old). The Student’s *T*-test was used to compare normally distributed continuous data, the Mann–Whitney *U*-test for non-normally distributed continuous data, and the chi-squared test for categorical data. Visualization using histograms and the Kolmogorov-Smirnov test were used to verify normality. OS per age group (<75 or ≥75 years old) was evaluated using the Kaplan–Meier method and compared using the log-rank test. All variables indicated in univariable analysis with a *P*-value < .1 were entered into a multivariable analysis using the (cause-specific) Cox proportional hazard regression model. The outcomes of the univariable and multivariable analyses were presented as hazard ratios (HR) along with 95% CI. A *P*-value of less than .05 was considered statistically significant. Since hazard ratios (HRs) from Cox regression do not account for competing events (such as death), competing risk regression was used to study disease recurrence. Death from any cause was used as competing event. Cumulative Incidence Function was plotted and compared using the Fine-Gray test. Missing data for key covariates were limited and multivariate analyses were performed using complete cases only.

Data analysis was performed using the Statistical Program for the Social Sciences (SPSS) version 28.0 (IBM Corp.) and STATA version 18.0.

## Results

### Patient enrolment

**Figure 1.**
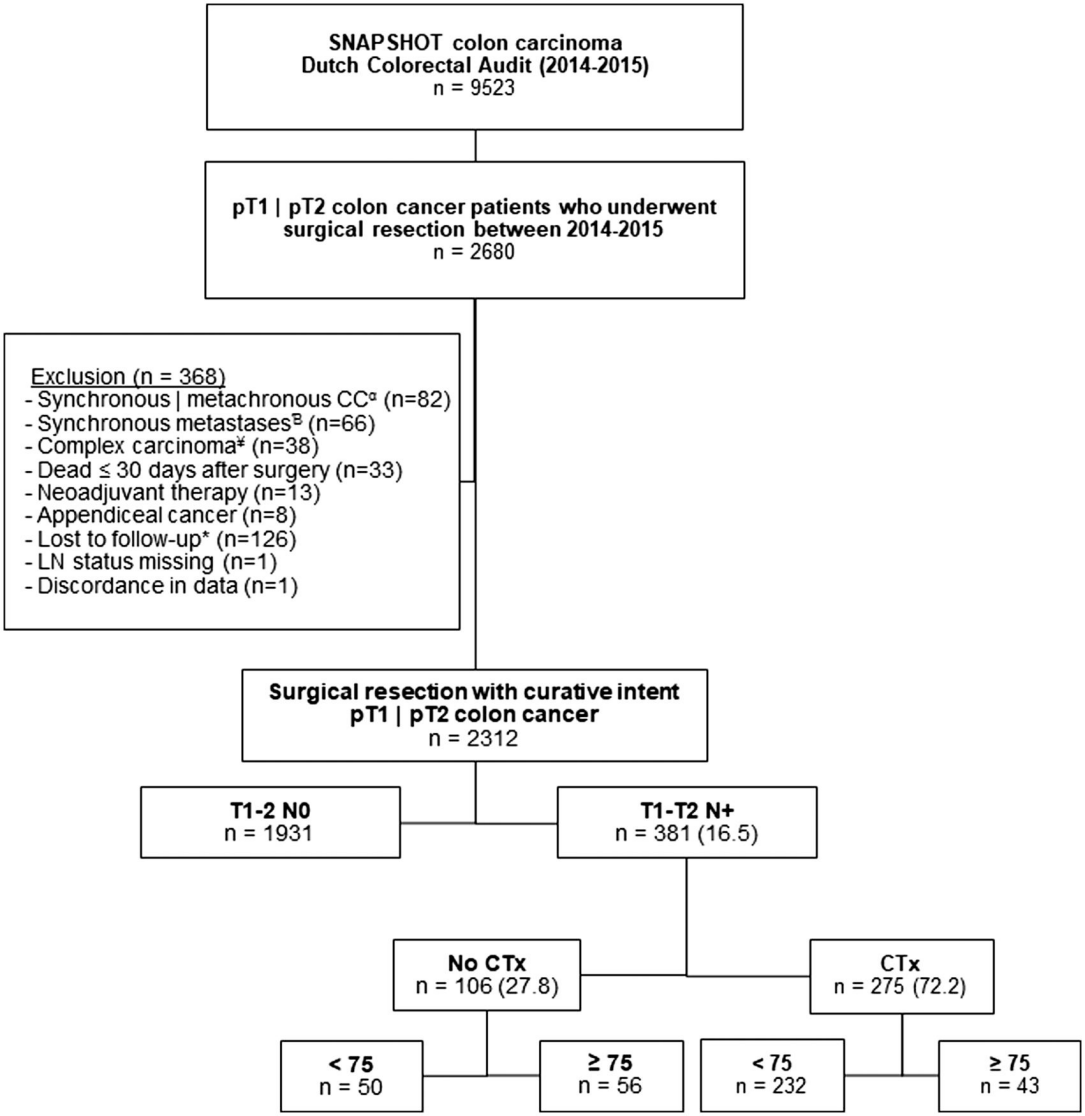
Flowchart patient enrollment. ^α^Synchronous colon cancer (multiple primary tumors in the resection specimen) or metachronous colorectal cancer (a second primary tumor occurring within 5 years after the first diagnosis). ^Ɓ^defined as the detection of distant metastasis either simultaneously with or within a 3-month interval of diagnosing the primary colon cancer. ^¥^defined as tumors with infectious tumor-related complications (proximal perforation, tumor perforation tumor, or peritumoral abscess) or clinical suspicion of ingrowth into surrounding structures or organs (cT4) based on preoperative imaging or intraoperative findings, for which a multivisceral resection was performed. ^*^Follow-up shorter than 3 months or missing follow up data.

The SNAPSHOT database comprised 9523 patients, of whom 2680 had undergone surgical resection for pT1 or pT2 CC. In total, 368 patients were excluded for predefined reasons (Figure 1). After exclusion, 2312 patients remained eligible for analysis. Among these patients, lymph node metastases (LNMs) were identified in 381 patients (16.5%), of whom 275 (72.2%) received adjuvant chemotherapy. The vast majority of this group consisted of patients aged under 75 years (*n* = 232). The group of patients who did not receive adjuvant chemotherapy (*n* = 106) included 50 patients under 75 years of age and 56 patients aged 75 years or older.

### Baseline characteristics

The baseline characteristics of all subgroups are presented in Table 1. In both age-stratified cohorts, patients who received adjuvant chemotherapy were significantly younger, and had lower ASA-scores. The median lymph node yield was similar in all groups. Among patients < 75 years, those who received chemotherapy had a higher proportion of pT2 patients (70.3% vs 54.0%, *P* = .03) and were more likely to have a lymph node ratio above 0.1 (58.2% vs 42.0%, *P* = .04).

**Table 1.** Baseline patient characteristics of patients diagnosed with pT1-2 N1-2 colon carcinoma stratified by age and administration of adjuvant chemotherapy (CTx).

	Age < 75 No CTx *n* = 50	Age < 75 CTx *n* = 232	*P* value	Age ≥ 75 No CTx *n* = 56	Age ≥ 75 CTx *n* = 43	*P* value
**Patient characteristics**						
**Age (years), mean ± SD**	68 ± 4.6	64 ± 7.0	**<.001**	80 ± 4.7	77 ± 1.9	**<.001**
**Sex, male, *n* (%)**	25 (50)	127 (54.7)	.54	26 (46.4)	19 (44.2)	.82
**ASA, *n* (%)**						
** I-II**	40 (80)	205 (88.4)	.11	33 (58.9)	37 (86)	**.003**
** III**	10 (20)	27 (11.6)		23 (41.1)	6 (14)	
**BMI (kg/m^2^), mean ± SD**	26.8 ± 5.3	27.6 ± 4.7	.28	27.1 ± 4.3	26.4 ± 3.9	.41
**Tumor location, *n* (%)**						
** Right colon**	21 (42)	163 (70.3)	.09	34 (60.7)	18 (41.9)	.06
** Left colon**	29 (58)	69 (29.7)		22 (39.3)	25 (58.1)	
**Surgical procedure, *n* (%)**						
** (Extended) right hemicolectomy**	19 (38)	57 (24.6)		34 (60.7)	15 (34.9)	
** Transversectomy**		2 (0.9)				
** (Extended) left hemicolectomy**	2 (4)	24 (10.3)		2 (3.6)	8 (18.6)	
** Sigmoid colectomy**	28 (56)	147 (63.4)		20 (35.7)	20 (46.5)	
** Subtotal colectomy**	1 (2)	2 (0.9)				
**Surgical approach, *n* (%)**						
** Laparoscopic**	41 (82)	198 (85.7)	.80	36 (65.5)	28 (65.1)	.70
** Open**	6 (12)	22 (9.5)		14 (25.5)	9 (20.9)	
** Conversion to open**	3 (6)	11 (4.8)		5 (9.1)	6 (14)	
**Post-operative complications, *n* (%)**	10 (20)	42 (18.1)	.75	18 (32.1)	12 (27.9)	.65
**Surgical complication, *n* (%)**	6 (12)	22 (9.5)	.59	9 (16.1)	8 (18.6)	.74
**Re-intervention,^a^ *n* (%)**	4 (8)	7 (3)	.10	5 (8.9)	3 (7)	.72
**Histological outcomes**						
**Lymph node yield, median [IQR]**	15 [11-19]	15 [11-20]	.60	15 [12-20]	14 [10-17]	.10
**Lymph node ratio**						
** <0.1**	29 (58)	97 (41.8)	**.04**	27 (48.2)	14 (32.6)	.12
** ≥0.1**	21 (42)	135 (58.2)		29 (51.8)	29 (67.4)	
**T-stage**						
** pT1**	23 (46)	69 (29.7)	**.03**	16 (28.6)	17 (39.5)	.25
** pT2**	27 (54)	163 (70.3)		40 (71.4)	26 (60.5)	
***N*-stage**						
** pN1**	36 (72)	195 (84.1)	**.04**	51 (91.1)	38 (88.4)	.66
** pN2**	14 (28)	37 (15.9)		5 (8.9)	5 (11.6)	
**Histological tumor type, *n* (%)**						
** Adenocarcinoma**	46 (93.9)	218 (95.6)	.72	50 (92.6)	41 (95.3)	.58
** Mucinous adenocarcinoma**	3 (6.1)	9 (3.9)		4 (7.4)	2 (4.7)	
** Signet ring cell carcinoma**		1 (0.4)				
** Unknown**	1 (2)	4 (1.7)		2 (3.6)		
**Tumor differentiation, *n* (%)**						
** Good/moderate**	45 (91.8)	207 (92)	.97	47 (87)	36 (87.8)	.91
** Poor**	4 (8.2)	18 (8)		7 (13)	5 (12.2)	
** Unknown**	1 (2)	7 (3)		2 (3.6)	2 (4.7)	
**Chemotherapy regimen**						
** CapOx**		198 (85.3)			15 (34.9)	
** Capecitabine**		17 (7.3)			21 (48.8)	
** FOLFOX**		2 (0.9)				
** Unknown**		15 (6.5)			7 (16.3)	

Abbreviations: ASA, American Society of Anesthesiologists physical status; BMI, body mass index.

a Defined as any invasive procedure (surgical, radiological or endoscopic) to treat a complication. Not included: central venous line or feeding tube <6 days, drainage of a superficial wound abscess on the ward, gastric siphon <6 days, urinary catheter <6 days.

In the chemotherapy group <75 years, the percentage of pN2 was lower compared to those who did not receive chemotherapy (15.9% vs 28.0%, *P* = .04). Additionally, the vast majority (85.4%) received CapOx, while in the elderly (≥ 75 years old), nearly half of the patients (48.8%) were treated with capecitabine monotherapy.

### Recurrence of disease

The rate of cancer recurrence and sites are summarized in Table 2. Among both age groups, the median follow-up of patients who did not receive chemotherapy was significantly shorter as compared to those who underwent adjuvant chemotherapy. In patients < 75 years, recurrence was higher in those who did not receive chemotherapy (16.0%) compared to those who did (8.6%), though the difference was not statistically significant (*P* = .11). Similarly, among patients aged ≥75, recurrence was more frequently seen in patients who were not treated with adjuvant chemotherapy (17.9% vs 7.0%), but this difference was also not significant (*P* = .11).

**Table 2.** Long-term oncological outcomes—stratified by age and administration of adjuvant chemotherapy (CTx).

	Total cohort No CTx *n* = 106	Total cohort CTx *n* = 275	*P* value	Age < 75 No CTx *n* = 50	Age < 75 CTx *n* = 232	*P* value	Age ≥ 75 No CTx *n* = 56	Age CTx *n* = 43	*P* value
**Follow-up (months), median [IQR]**	48 [24-60]	62 [57-68]	**<.001**	50 [25-60]	62 [58-69]	**<. 001**	45 [23-60]	57 [48-62]	**.01**
**Recurrent disease, n (%)**	18 (17)	23 (8.4)	**.02**	8 (16)	20 (8.6)	.11	10 (17.9)	3 (7)	.11
** Loco regional recurrence only**	3 (2.8)	1 (0.4)		1 (2)	1 (0.4)		2 (3.6)	0	
** Distant metastases only**	14 (13.2)	16 (5.8)		7 (14)	13 (5.6)		7 (12.5)	3 (7)	
** Loco regional recurrence + distant metastases**	1 (0.9)	6 (2.2)		0	6 (2.6)		1 (1.8)	0	
**Loco regional recurrence, n (%)**	4 (3.8)	8 (2.9)	.67	1 (2)	8 (3.4)	.60	3 (5.4)	0	.12
** Anastomosis**		1			1				
** Regional lymph nodes**	1	1			1		1		
** Peritoneum**	3	5		1	5		2		
** Multifocal**		1			1				
**Distant metastases, n (%)**	15 (14.2)	22 (8)	.07	7 (14)	19 (8.2)	.20	8 (14.3)	3 (7)	.25
** Liver**	12	17		5	16		7	1	
** Lung**	8	10		5	8		3	2	
** Non-regional lymph nodes**	2	4		2	3			1	
** Other**	5	8		1	7		4	1	
**5-year overall survival (%)**	60.9	90.3	**<.001**	68.1	91.3	**< .001**	55.1	84.5	**.003**
**5-year cumulative incidence of recurrence^a^ (95% CI)**	0.18 (0.11-0.27)	0.09 (0.06-0.12)	**.007**	0.18 (0.08-0.31)	0.09 (0.06-0.13)	.06	0.18 (0.14-0.31)	0.07 (0.02-0.17)	.10

a Fine and Gray analysis with using death from any cause as competing event.

**Figure 2.**
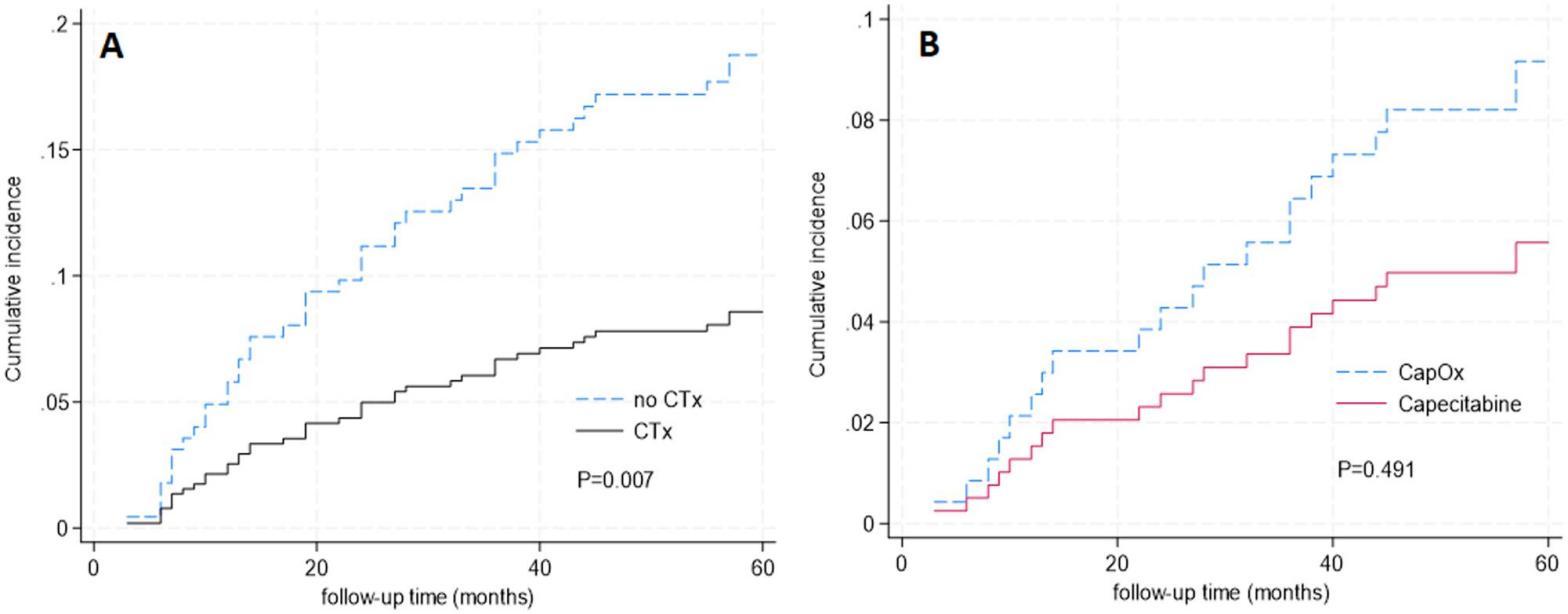
(A) Cumulative incidence function of 5-year recurrence in the total cohort with death as competing event. (B) Cumulative incidence function of 5-year recurrence of patients receiving CapOx vs Capecitabine monotherapy with death as competing event.

In general, the proportion of recurrence was significantly lower in patients who received adjuvant chemotherapy (8.4%) compared to those who did not (17.0%) (*P* = .02), with a cumulative incidence of recurrence (CIF) of 0.18 in the CT- group (95% CI 0.11–0.27) vs 0.09 (95% CI 0.06-0.12) in the CT+ group (Figure 2A). There were no factors associated with recurrence of disease, other than administration of adjuvant chemotherapy (Table 3). The cumulative incidence of recurrence did not differ between patients receiving CapOx or Capecitabin monotherapy (CIF 0.09, 95% CI 0.06-0.14 vs 0.05 95% CI 0.01-0.16, Fine-Gray *P* = .49) (Figure 2B).

**Table 3.** Cause-specific Cox proportional hazard and competing risk regression analyses of factors associated with 5-year recurrence.

		Cause-specific Cox proportional hazard		Competing risks regression	
Prognostic factor	No. of patients	Unadjusted HR (95% CI)	Adjusted HR (95% CI)	Unadjusted SHR (95% CI)	Adjusted SHR (95% CI)
**Age group**					
** < 75**	282	ref	ref	ref	ref
** ≥ 75**	99	1.4 (0.7-2.7)	0.8 (0.4-1.7)	1.4 (0.7-2.6)	0.8 (0.3-1.7)
**Tumor location**					
** Left colon**	239	ref	ref	ref	ref
** Right colon**	142	1.6 (0.9-2.9)	1.3 (0.6-2.6)	1.5 (0.8-2.8)	1.2 (0.6-2.5)
**Lymph node yield**					
** ≥ 12**	274	ref	ref	ref	ref
** < 12**	107	0.7 (0.3-1.5)	0.7 (0.3-1.7)	0.7 (0.3-1.5)	0.7 (0.3-1.8)
**Lymph node ratio**					
** < 0.1**	167	ref	ref	ref	ref
** ≥ 0.1**	214	1.2 (0.6-2.2)	1.7 (0.8-3.8)	1.2 (0.6-2.2)	1.7 (0.8-3.7)
**T-stage**					
** pT1**	125	ref	ref	ref	ref
** pT2**	256	1.3 (0.7-2.7)	1.2 (0.6-2.6)	1.3 (0.7-2.7)	1.2 (0.6-2.6)
				F	
**N-stage**					
** pN1**	320	ref	ref	ref	ref
** pN2**	61	1.4 (0.6-2.9)	1.1 (0.5-2.6)	1.3 (0.6-2.8)	1.1 (0.5-2.5)
**Tumor differentiation**					
** Good/moderate**	335	ref	ref	ref	ref
** Poor**	34	0.8 (0.2.-2.5)	0.6 (0.2-2.1)	0.8 (0.2-2.6)	0.6 (0.2-2.3)
**Adjuvant chemotherapy**					
** No**	106	ref	ref	ref	ref
** Yes**	275	**0.4 (0.2-0.8)**	**0.3 (0.2-0.7)**	**0.4 (0.2-0.8)**	**0.3 (0.2-0.7)**
**Chemotherapy regimen**					
** CapOx**	213	ref	ref	ref	ref
** Capecetabine monotherapy**	38	0.6 (0.1-2.6)	0.3 (0.04-3.2)	0.6 (0.1-2.6)	0.3 (0.05-2.5)

Adjusted for age group, tumor location, lymph node yield, lymph node ratio, T-stage, N-stage, and tumor differentiation.

### Overall survival of patients for different age groups

**Figure 3.**
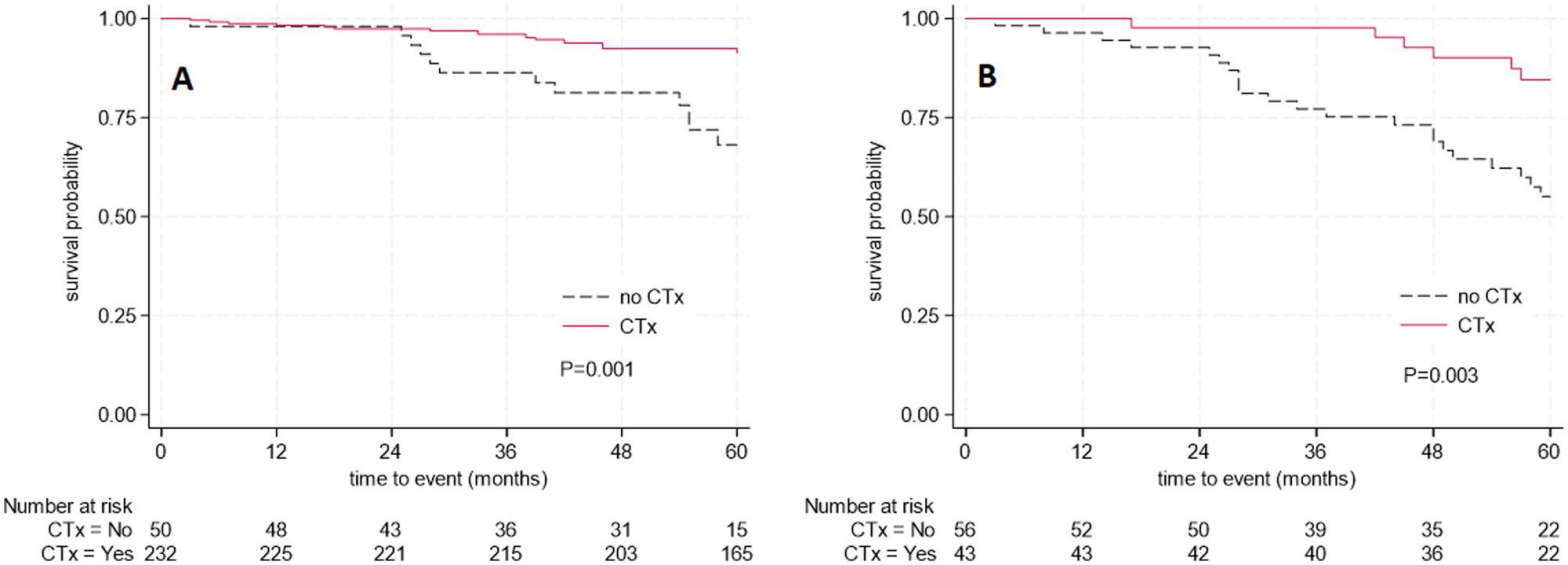
(A) Five-year overall survival pT1-2N+ patients with colon cancer younger than 75 years old, with and without adjuvant chemotherapy (CTx). (B) Five-year overall survival pT1-2N+ patients with colon cancer ≥ 75 years old, with and without adjuvant chemotherapy (CTx).

Patients < 75 years old who did not receive chemotherapy exhibited a significantly lower 5-year OS rate: 68.1% vs 91.3%, log-rank *P* < .001 (Figure 3A). After adjustment for age, ASA score, tumor location, occurrence of postoperative complications, and N stage, the administration of chemotherapy remained independently associated with OS, with an adjusted HR of 0.3 (95% CI 0.1-0.7) (Table S1).

Patients ≥ 75 years who did not receive chemotherapy had a significantly lower 5-year OS rate of 55.1%, compared to 84.5% for those who did receive chemotherapy (*P* = .003) (Figure 3B). However, this effect was not statistically significant anymore after adjusting for age and BMI, with an adjusted hazard ratio (HR) for adjuvant chemotherapy of 0.5 (95% CI 0.2-1.3) (Table 4). The chosen chemotherapy regimen (CapOx or Capecitabine monotherapy) was also not found to be significantly associated with OS (HR 0.8, 95% CI 0.2-3.9, *P* = .76).

**Table 4.** Univariable and multivariable analyses of factors associated with 5-year overall survival in patients ≥ 75.

Prognostic factor	No. of patients	Univariate HR (95% CI)	*P* value	Multivariate HR (95% CI)	*P* value
**Age (years), continuous**	99	1.13 (1.1-1.2)	**< .001**	1.2 (1.1-1.3)	**< .001**
**BMI (kg/m^2^)**	99	0.91 (0.8-1.0)	**.08**	0.9 (0.8-1.0)	.07
**Sex**					
** Male**	45	ref			
** Female**	54	1.4 (0.6-3.0)	.40		
**ASA**					
** I-II**	70	ref			
** III-IV**	29	1.5 (0.7-3.2)	.35		
**Tumor location**					
** Left colon**	47	ref			
** Right colon**	52	1.6 (0.8-3.5)	.21		
**Post-operative complications**					
** No**	69	ref			
** Yes**	30	1.0 (0.4-2.3)	1.00		
**Surgical complication**					
** No**	82	ref			
** Yes**	17	0.3 (0.1-1.4)	.14		
**Lymph node yield, continuous**	99	1.02 (0.9-1.1)	.43		
** ≥ 12**	70	ref			
** < 12**	29	0.5 (0.2-1.2)	.11		
**Lymph node ratio**					
** < 0.1**	41	ref			
** ≥ 0.1**	58	0.9 (0.4-1.9)	.73		
**T-stage**					
** pT1**	33	ref			
** pT2**	66	1.8 (0.7-4.1)	.20		
**N-stage**					
** pN1**	89	ref			
** pN2**	10	1.3 (0.4-3.7)	.65		
**Tumor differentiation**					
** Good/moderate**	83	ref			
** Poor**	12	1.1 (0.4-3.1)	.89		
**Adjuvant chemotherapy**					
** No**	56	ref		ref	
** Yes**	43	0.3 (0.1-0.7)	**.005**	0.5 (0.2-1.3)^a^	.14
**Chemotherapy regimen**					
** CapOx**	15	ref			
** Capecetabine monotherapy**	21	0.8 (0.2-3.9)	.76		

a Adjusted for age and BMI.

## Discussion

In this multicenter, retrospective real-world cohort study, the benefits of adjuvant chemotherapy were assessed in patients with node-positive pT1-2 colon cancer, stratified by age group. While the rate of cancer recurrence was higher in patients who did not receive chemotherapy compared to those who did, this difference was not statistically significant in either age group. However, across the entire cohort, patients who received adjuvant chemotherapy had a significantly lower rate of recurrence. In patients under 75 years, adjuvant chemotherapy was significantly associated with improved overall survival, while in those aged 75 or older, no significant survival benefit could be demonstrated after adjusting for confounding factors.

To the best of our knowledge, this study represents the largest population of patients with lymph node-positive pT1 and pT2 colon cancer ever examined to assess the potential benefits of chemotherapy on overall survival and risk of recurrence. Pian et al. observed that overall- and disease-free survival was similar between patients with pT1-2 N1 CC with and without chemotherapy, and even compared to patients without synchronous lymph node metastases (pT1-2 N0), but with very small patient numbers (*n* = 16 per chemotherapy group).^8^ Kim et al. studied a cohort with the same magnitude as ours and found no effect of chemotherapy on recurrence-free survival. However, the number of patients who did not undergo chemotherapy was small (*n* = 39), overall survival was not assessed, and it is unclear whether competing events, such as death from other causes, were accounted for. Furthermore, the group of patients who did not undergo chemotherapy, which was used as a reference group, did include patients who received oral chemotherapy with pyrimidine analogues or oral 5-FU, which could have affected their results.^10^ Although our cohort is larger than previously reported, it may still be underpowered to detect differences in disease recurrence, as differences between patients receiving chemotherapy and those who did not were not statistically significant within separate age groups but became significant when the entire cohort was analyzed together.

A pooled analysis of 7 randomized clinical trials involving 500 patients older than 70 years of age, showed that effects of adjuvant treatment on survival were comparable across all ages.^17^ The studies incorporated in this analysis originate from the 1980s and used outdated chemotherapy schemes. Furthermore, the participants enrolled in these clinical trials represented a selected patient population characterized by a more favorable performance status, with only a certain degree of comorbidities and a limited age range, with 85% being under 70 years old. More recently, a pooled analysis of 12 trials compared the effects of adjuvant chemotherapy in patients younger than 70 years (*n* = 13 569) and those older than 70 years (*n* = 4340) found that time to recurrence was similar for both age groups receiving chemotherapy.^18^ However, patients aged over 70 seemed to benefit less from treatment than their younger counterparts. They demonstrated lower DFS, cancer-specific survival, and OS, along with higher rates of early treatment discontinuation and toxicity.^18^ Nevertheless, it is important to note that fewer than 10% of these patients were diagnosed with pT1 or pT2 colorectal cancer, and all were sufficiently healthy to participate in clinical trials, making it difficult to extrapolate these findings to daily practice.

In contrast, various “real-world” retrospective cohort studies criticize the use of systemic adjuvant treatment in older patients, aligning with our findings.^19^^,^^20^ In these studies, patients ≥ 75 did not benefit from adjuvant chemotherapy. However, in the study by Liu et al., the proportion of pT1-2 patients was again rather low (5%). Besides the absolute survival gain that adjuvant chemotherapy is offered to older patients, there is controversy about the benefit of adding oxaliplatin to a fluoropyrimidine in elderly patients. Various studies that compared CapOx to Capecitabine monotherapy in elderly, showed that both schemes provide an equal amount of survival benefit, which is in line with our results.^21–25^ However, these studies used different cut off values of (older) age and analyzed study populations with once again a small proportion or not any pT1-2 patients.

It is highly questionable whether well-substantiated evidence on the added value of adjuvant chemotherapy in patients with pT1-2N1-2 CC will ever be provided. Studying this in a randomized setting is likely not feasible. The risk of lymph node metastases at baseline is already very low in this patient category, as are rates of recurrent disease, making it difficult to achieve the required sample sizes. In retrospective real-world studies, such as the present study, a significant issue remains: the high degree of selection and allocation biases. This is reflected by the analyses of younger patients, where patients receiving chemotherapy group did not show significant lower rates of relapse but did demonstrate higher overall survival and the major difference in the OS of patients ≥ 75 years old (84.5% vs 55.1%). This discrepancy likely reflects differences in frailty and comorbidity within the patient population that cannot be adjusted solely based on age and ASA score. Given the much lower OS compared to the rate of recurrent disease in the patients not receiving adjuvant chemotherapy across both age groups, other-cause mortality is likely higher in patients not receiving adjuvant chemotherapy, rather than colon cancer-related deaths. Considering these limitations, overall survival may be less informative, and although disease recurrence can provide valuable insight, colorectal cancer–specific mortality would be a more appropriate endpoint, but unfortunately it was not recorded for patients in this cohort. The study is also limited by missing data on chemotherapy completion, dose reductions, and treatment-related toxicity. Therefore, the lack of a significant association between adjuvant chemotherapy and recurrence or survival in some subgroups could reflect treatment interruptions or dose reductions due to toxicity rather than a true absence of effect. Furthermore, the small and imbalanced groups render the study underpowered to determine whether Capecitabine monotherapy is non-inferior to CapOx, as reflected by the wide confidence intervals. At last, we need to recognize that immortal time bias may constitute a limitation of our study. Patients in the control group who did not receive adjuvant chemotherapy may have been at higher risk of early mortality, potentially resulting in a shorter observed follow-up duration in this group. Such an imbalance could lead to an overestimation of the apparent survival benefit associated with adjuvant chemotherapy. To evaluate this potential bias, we conducted sensitivity analyses of patients with limited follow up (>24 months). No statistically significant differences were found in all-cause mortality between patients who received chemotherapy and those who did not.

In conclusion, we demonstrated that adjuvant chemotherapy was associated with a reduced risk of disease recurrence in patients with node-positive pT1-2 colon carcinoma, regardless of age group stratification. However, the association of adjuvant chemotherapy with overall survival appears age-dependent, showing benefit in patients under 75 but no significant association with improved survival in those aged ≥75, regardless of the chemotherapy regimen. These findings highlight the need for individualized treatment strategies based on patient age and overall prognosis. Overall, the strength of the evidence supporting the effectiveness of adjuvant chemotherapy in patients with pT1N+ and pT2N+ CC remains questionable. Landmark studies have either excluded pT1 and pT2 patients or included very few of them, and retrospective data inherently carry significant selection and allocation biases, which are challenging to address. Although this study represents the largest pT1-2N+ cohort analyzed to date, the sample size remains small due to the low incidence of lymph node metastases in pT1 and pT2 carcinomas. It is crucial for clinicians to be aware that the evidence supporting adjuvant chemotherapy for this patient population is weak, particularly for the elderly. This should be taken into account during discussions about treatment options with patients.

## Supplementary Material

oyag171_Supplementary_Data

## Data Availability

The datasets generated during and/or analyzed during the study are not publicly available but are available from the corresponding author on reasonable request.
