# Supplementary material for: Added value of adjuvant chemotherapy in patients with node-positive pT1-2 colon cancer: a national SNAPSHOT analysis
Source: Oncologist. 2026 May 13;31(6):oyag171. doi: 10.1093/oncolo/oyag171 (PMC13192554; doi:10.1093/oncolo/oyag171)
Supplement: oyag171_Supplementary_Data [file oyag171_supplementary_data.docx]

**Table S1. Univariable and multivariable analysis of factors associated with 5-year overall survival in patients < 75**

* Adjusted for age, ASA, tumor location, postoperative complications and N-stage

| Prognostic Factor | No. of patients | Univariate HR (95% CI) | P value | Multivariate HR (95% CI) | P value |
| --- | --- | --- | --- | --- | --- |
|  |  |  |  |  |  |
| Age (years), continuous | 282 | 1.1 (0.9-1.1) | **0.08** | 1.0 (0.9-1.1) | 0.34 |
| BMI (kg/m^2^) | 282 | 1.0 (0.9-1.1) | 0.59 |  |  |
| Sex |  |  |  |  |  |
| Male | 152 | ref |  |  |  |
| Female | 130 | 1.7 (0.8-3.5) | 0.14 |  |  |
|  |  |  |  |  |  |
| ASA |  |  |  |  |  |
| I-II | 245 | ref |  | ref |  |
| III-IV | 37 | 3.0 (1.4-6.6) | **.005** | 2.5 (1.1-5.5) | **0.02** |
|  |  |  |  |  |  |
| Tumor location |  |  |  |  |  |
| Left colon | 192 | ref |  | ref |  |
| Right colon | 90 | 3.3 (1.6-6.7) | **.001** | 2.7 (1.3-5.6) | **0.01** |
|  |  |  |  |  |  |
| Post-operative complications |  |  |  |  |  |
| No | 230 | ref |  | ref |  |
| Yes | 52 | 2.5 (1.2-5.3) | **0.01** | 1.9 (0.9-4.2) | 0.10 |
|  |  |  |  |  |  |
| Surgical complication |  |  |  |  |  |
| No | 254 | ref |  |  |  |
| Yes | 28 | 1.8 (0.7-4.7) | 0.23 |  |  |
|  |  |  |  |  |  |
| Lymph node yield , continuous | 282 | 1.0 (0.9-1.0) | 0.99 |  |  |
| ≥ 12 | 204 | ref |  |  |  |
| < 12 | 78 | 0.9 (0.4-2.0) | 0.82 |  |  |
|  |  |  |  |  |  |
| Lymph node ratio |  |  |  |  |  |
| < 0.1 | 126 | ref |  |  |  |
| ≥ 0.1 | 156 | 0.7 (0.4-1.5) | 0.40 |  |  |
|  |  |  |  |  |  |
| T-stage |  |  |  |  |  |
| pT1 | 92 | ref |  |  |  |
| pT2 | 190 | 0.8 (0.4-1.8) | 0.64 |  |  |
|  |  |  |  |  |  |
| N-stage |  |  |  |  |  |
| pN1 | 231 | ref |  | ref |  |
| pN2 | 51 | 2.4 (1.2-5.1) | **0.02** | 2.1 (0.9-4.6) | 0.06 |
|  |  |  |  |  |  |
| Tumor differentiation |  |  |  |  |  |
| Good/moderate | 252 | ref |  |  |  |
| Poor | 22 | 0.7 (0.2-3.0) | 0.67 |  |  |
|  |  |  |  |  |  |
| Adjuvant chemotherapy |  |  |  |  |  |
| No | 50 | ref |  |  |  |
| Yes | 232 | 0.3 (0.1-0.5) | **< .001** | 0.3 (0.1-0.7)* | **.002** |
